# Heterogeneity of Signal Transducer and Activator of Transcription Binding Sites in the Long-Terminal Repeats of Distinct HIV-1 Subtypes

**DOI:** 10.2174/1874357900701010026

**Published:** 2007-10-20

**Authors:** Andrea Crotti, Giulia D. Chiara, Silvia Ghezzi, Rossella Lupo, Rienk E Jeeninga, Elio Liboi, Patricia M.-J Lievens, Elisa Vicenzi, Chiara Bovolenta, Ben Berkhout, Guido Poli

**Affiliations:** 1 AIDS Immunopathogenesis, San Raffaele Scientific Institute, Milano, Italy; 2 Viral Pathogens and Biosafety Units, San Raffaele Scientific Institute, Milano, Italy; 3 MolMed SpA, Milano, Italy; 4 Laboratory of Experimental Virology, Center for Infection and Immunity Amsterdam (CINIMA), Academic Medical Center, University of Amsterdam, Amsterdam, The Netherland; 5 Division of Biochemistry, Department of Morphological and Biomedical Sciences, University of Verona Medical School, Verona, Italy; 6 Vita-Salute San Raffaele University, School of Medicine, Milano, Italy

**Keywords:** STAT5, HIV, LTR, virus subtype, cytokines, transcription.

## Abstract

HIV-1 can be subdivided into distinct subtypes; the consequences of such a genomic variability remain largely speculative. The long terminal repeats (LTR) control HIV transcription and reflect the major differences of distinct viral subtypes. Three regions in the HIV-1 subtype B LTR are close matches to the Signal Transducer and Activator of Transcription (STAT) consensus sequence. Here, we show heterogeneity in these putative STAT binding sites among HIV-1 LTR subtypes A through G. Transfection of constitutively activated STAT5 lead to transcriptional activation of HIV-1 expression in 293T cells transfected with a reporter assay driven by HIV-1 LTR subtype B. Constitutively activated STAT5 transactivated the LTR of various subtypes in U937 cells with different potency. These findings support and expand the potential relevance of STAT5 activation in HIV infection and may bear relevance for a differential regulation of latency and expression of different subtypes of HIV-1.

## INTRODUCTION

The human immunodeficiency virus type 1 (HIV-1), the etiological agent of acquired immunodeficiency syndrome (AIDS), can be classified in 3 distinct groups defined as major (M), outlier (O) and new (N). Most HIV-1 isolates identified to date in the pandemic belong to the group M that has spread worldwide within the last 25 years [1]. The other two HIV-1 groups are confined to a more restricted geographical area in Sub-Saharan Africa whereas several infected individuals from West-Central Africa harbor viruses from the distinct group O. More recently, one member of the third group N was isolated from an AIDS patient in Cameroon [2]. Group M viruses, responsible for the global pandemic, have diversified during their worldwide spreading and have been grouped according to their genomic sequences leading to at least 10 distinct subtypes (or clades) termed A through K [1, 3]. These different subtypes are not distributed evenly in that subtype B predominates in North America and Europe, whereas subtype C prevails in Sub-Saharan Africa [4]. Further, more than 20 Circulating Recombinat Forms (CRF) have been reported [3]. The relevance of CRFs in the global HIV-1 pandemics is increasingly recognized, accounting for 18% of incident infecion [5, 6] and representing the local predominant form in Southeast Asia (CRF01-AE) [7, 8] or in West and West-Central Africa (CRF02-AG) [9, 10]. At present, there is no clear-cut evidence for subtype specific variation in virulence or transmission, and their diverse geographical distribution is likely to result from stochastic founder effects. Nevertheless, the possibility that the different subtypes are heterogeneous in some aspects of their biological properties, such as cell tropism and/or regulation of gene transcription, which may affect their pathogenic potential, cannot be excluded. In this regard, the non-coding HIV-1 long terminal repeats (LTR), the major transcriptional regulator unit of virus expression, reflect the major differences observed among HIV-1 subtypes. For example, both duplications and diminution of DNA binding sites for the cellular transcription factor NF-kB have been described in the case of clade C and clade AE (CRF-01-AE), respectively [11-14].

In this scenario, 3 independent regions in the HIV-1 LTR subtype B have been recently identified as close matches to the signal transducer and activator of transcription 5 (STAT5) consensus-binding sequence along with functional evidence of an upregulatory effect of STAT5 activation on viral transcription [15]. STAT5 is a transcription factor triggered by several type I cytokines either belonging to the γ-common (γc, i.e. interleukin-2, IL-2, IL-7, IL-9, IL-15, IL-21) or βc (IL-3, IL-5 and granulocyte-macrophage colony stimulating factor, GM-CSF) families [16, 17]. Thus, STAT5 represents a key factor transducing the effects of several cytokines and its involvement in the regulation of HIV-1 subtype B transcription raises the question about its potential role in controlling the expression of other HIV-1 subtypes.

Therefore, in the present study we analyzed the LTR sequences of different HIV-1 subtypes in order to verify the presence and potential variability of putative STAT-binding sites and investigated their role in the regulation of viral transcription. We indeed report the presence of heterogeneity in such putative STAT binding sites among the different HIV-1 LTR subtypes A through G, including an AG CRF. In addition, we provide direct evidence of the inductive role of constitutively phosphorylated STAT5 in triggering HIV-1 LTR subtype B transactivation, in the absence of cytokine stimulation. Finally, we demonstrate that constitutively phosphorylated STAT5 transactivates HIV-1 LTR subtype A through G with different potency.

## MATERIALS AND METHODS

### Cell lines and reagents.

HEK 293T cells were propagated in I-MDM (Bio-Whittaker, Verviers, Belgium) supplemented with 10% FCS (Bio-Wittaker) and penicillin-streptomycin-glutamine (PSG). The U937 cell line was maintained in RPMI 1640 medium (Bio-Whittaker), containing 10% FCS and PSG. Recombinant granulocyte-macrophage colony stimulating factor (GM-CSF, R&D Systems, Minneapolis, MS) was used at 20 ng/ml, based on previous results [18].

### LTR nucleotide sequence analysis.

Specific subtypes LTR sequence were obtained and characterized as reported in Jeeninga *et al.*, 2000 [14]. Briefly, human serum samples from patients suspected of having a non-subtype B HIV-1 infection were selected from the outpatient clinic of the Academic Medical Center of the University of Amsterdam (Amsterdam, The Netherlands), and the LTR-*gag *region of the viral genome was amplified by reverse transcription (RT)-PCR as described [19]. A detailed comparison of these viral sequences with their subtype reference sequences has been discussed previously [19]. The 3’ HIV-LTR nucleotide sequences of the subtype A through G and G” (CRF-AG) [14] were analyzed for the identification of potential transcription factor binding sites with two independent softwares: MatInspector Professional (Genomatix Software, Munich, Germany, http://www.genomatix.de/index.html; date of access: 03-18-05), based on the MatInspector program [20] using the selected matrix library (vertebrate section) and optimized thresholds, and TFSEARCH (www.rwcp.or.jp/papia; date of access: 03-10-05), based on COMPEL databases (www.transfac.gbf.de/TRANSFAC or www.bionet.nsc.ru/TRRD).

### Nucleotide sequence accession numbers.

LTR nucleotide sequences from representative subtype clones have been deposited in the GenBank database. Their accession numbers are: AF127566 (subtype A), AF127567 (subtype C), AF127569 (subtype D), AF127570 (subtype E (CRF01-AE)), AF127571 (subtype F), AF127572 (subtype G), and AF127573 (subtype G”(CRF02-AG)). Strain specific sequences for intra-subtype heterogeneity analysis were obtained from Los Alamos HIV Database (http://www.hiv.lanl.gov/content/hiv-db).

### Plasmids.

The HA-tagged STAT5A c-DNA (kindly donated by B. Mathey-Prevot, Harvard University, Boston, MA) was subcloned into the expression vector pXM to generate the pXM-HA-STAT5A plasmid. The N642H mutation, that renders STAT5 constitutively phosphorylated [21, 22], was created by PCR mutagenesis as described [18]. The pGL2-β-Casein-luciferase (luc) construct contains 4 tandem repeats GAS sequences from the murine casein promoter (core sequence: ATTTCTAGGAAATCG) inserted upstream of the luc gene in a pGL2 vector (Promega) [23]. The generation and characterization of the pBlue3’LTR-luc plasmids, containing LTR sequence from different subtypes, has been previously described [14]. The eGFP-PΔN lentiviral vector was obtained by inserting the eGFP PCR-amplified ORF in the *Cla*I site upstream the PGK-ΔLNGFR selection marker cassette in the HIV-1 based lentiviral vector PΔN vector as described [24].

### Pseudotyped lentiviral vector production, transduction, ΔLNGFR immune selection and LTR-GFP assay.

VSV-G pseudotyped PΔN-GFP lentiviral vector stock production, the transduction of 293T cells with the corresponding vector, and the following LNGFR immune selection to >95% purity, were performed following standardized procedures as reported in [24]. NGFR^+^ transduced cells showing a constitutive basal GFP expression (NGFR^+^/GFP^+^) were sorted to obtain a highly enriched NGFR^+^/GFP^- ^subpopulation. Cells were then transfected with different amounts of pXM or pXM-HA-STAT5A or pXMHA-STAT5A-P (expressing constitutively activated STAT5A) by Fugene6^®^ according to the manufacturer’s instructions (Roche, Indianapolis, IN). For detection of LTR-driven GFP expression, cells were acquired by FACScan^®^ (Becton Dickinson, Franklin Lakes, NJ) and analyzed by CellQuest software (Becton Dickinson).

### Transfection and luc activity assay.

U937 cells were transfected by the Amaxa electroporator Nucleofector I and Nucleofector Kit V (program V-01) according to the manufacturer’s procedure (AMAXA Biosystems, Cologne, Germany). In order to evaluate transfection efficiency (estimated to be approximately 50%), cells were transfected with the pmaxGFP plasmid (AMAXA Biosystems). STAT5-induced activation of LTR-luc construct (pBlue3’LTR-Luc) was determined by co-transfection with either pXM, pXM-HA-STAT5A, or pXM-HA-STAT5A-P. Twenty-four h after transfection, cell lysates were mixed with the luciferin substrate (Promega, Madison, WI) and luc activity was measured by a luminometer (Lumino, Stratec Electronic, Bath, UK). STAT5-activated LTR activity was calculated as relative luc units (RLU)/mg protein of the lysate and expressed as fold increase above basal level.

### Electrophoretic mobility shift assay (EMSA).

Whole cell extracts (WCE) were prepared as previously described [25]. WCE were incubated with different [γ-^32^P]-ATP-end labeled double stranded oligonucleotides corresponding to the STAT binding consensus sequences (Santa Cruz Biotechnology, Inc., Santa Cruz, CA) or the STAT-binding sequence of HIV-1 subtype G LTR (Fwd 5’- GGA CTT TCC GGG AAG CCC CGC C - 3’; Rev 5’- GGC GGG GCT TCC CGG AAA GTC C -3’) as described [25].

## RESULTS

### Heterogeneous putative STAT DNA binding sequences in the LTR of different HIV-1 subtypes.

In order to investigate the potential role of STAT5 as modulator of HIV transcription and virus expression, we have searched for putative STAT-binding sites in the HIV-1 LTR by the Genomatrix software. To this aim we analyzed specific subtypes LTR sequences previously obtained and characterized by Jeeninga *et al.* 2000 [14]. The subtype A sequence was actually obtained from an individual infected with an AC CRF with the LTR element derived from subtype A[14]. The CRF01-AE is now the more accepted sequence representing HIV-1 subtype E with the LTR portion from subtype E since no full length E viral isolate has been obtained thus far [7, 8]. The subtype G” is a cluster of sequences from AG CRF (CRF-IbNG) with the LTR portion that is closely related to that of subtype G [26].

We identified a region, located between -77 and -85 in the LTR matching to the STAT-consensus binding sequence, i.e.: 5’-TTC (N3) GAA-3’, as shown in Fig. (**1**). This putative consensus STAT binding site showed different degrees of homology in the LTR of subtypes A through G and G” (CRF02-AG) (Table **1**).

**Fig. (1):**
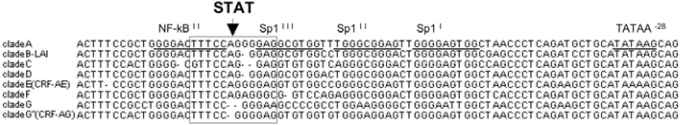
**STAT putative binding site are present in the LTR of different HIV-1 subtypes**. Alignment of the HIV 3’LTR sequences of subtype A through G and G”: putative STAT DNA binding sites are boxed. Underlined are NF-κB and Sp1 binding sites. Reference STAT5 binding consensus sequence: TTC (N3) GAA [46].

All the subtypes showed a conserved 5’ sequence TTC, whereas several differences were observed both in the length of the spacer (N3) region and in the 3’ sequence GAA (Fig. **1**). The canonical 3 nucleotide length of this spacer region was conserved only in subtypes C, G and G”(CRF02-AG), whereas 4 nucleotides were present in subtypes B and D and a longer spacer (5 nucleotides) was observed in subtypes A, E (CRF01-AE) and F (Fig. **1**). The 3’ sequence GAA was conserved only in subtype G in that all the other subtypes showed either an A-to-C or an A-to-G substitution at the level of the third nucleotide of the 3’ sequence. Finally, an additional A-to-G substitution was observed at the level of the second nucleotide of the subtype F and G”(CRF02-AG) 3’ sequence (Fig. **1**). Of interest is the fact that the subtype G sequence perfectly matched the STAT binding consensus sequence. Subtypes B, C and D putative STAT-binding sequences LTR contained regions with a similar degree of homology to the canonical STAT binding consensus sequence, whereas a lower score of matching was assigned to the sequence present in the LTR of subtype A and E (CRF01-AE). Finally, subtype F showed the lowest homology and was actually under the threshold of the assay (Table **1**). Similar results were obtained using TFSearch, an independent bioinformatic software analyzing the presence of putative binding sites for transcription factors (data not shown). Each of the eight subtypes LTR sequences analyzed for the presence of STAT binding site was then aligned with strain specific sequences from the Los Alamos HIV Database (http://www. hiv.lanl.gov/content/hiv-db), in order to evaluate their heterogeneity in this small nucleic acid motif. All subtype prototypical sequences were representative of more than 50% of the sequences deposited in the Database, with the exception of subtype G (Table **2**). In this regard, most (67.8%) of the clade G STAT binding sites were identical to the sequence found in clade G”(CRF02-AG) (data not shown). The STAT binding sequence of the subtype B-LAI virus was indicated as a prototypical "B" site in that conserved in >60% of the sequences present in the Los Alamos Database and because it was previously adopted as reference sequence [15]. These findings support and extend the observation that a consensus STAT binding element is present in the subtype B HIV-1 LTR [15].

### Constitutively phosphorylated STAT5 triggers subtype B HIV-1 LTR activation.

**Fig. (2):**
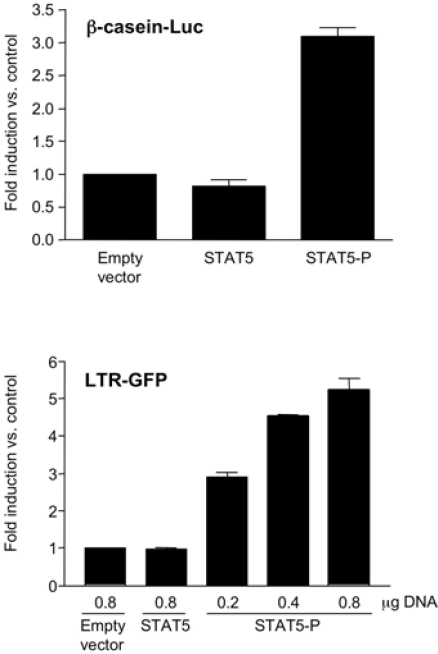
**STAT5–P, but not STAT5, activates the subtype B HIV-1 LTR**. 293T cells were co-transfected with equal amounts of a β-casein responsive firefly luc reporter construct (upper panel) and either an empty vector or vectors expressing STAT5 or STAT5-P. Similar constructs were transfected in 293T-LTR-GFP cells (see Material & Methods). The results represent the mean ± SEM of 3 independent experiments.

**Fig. (3):**
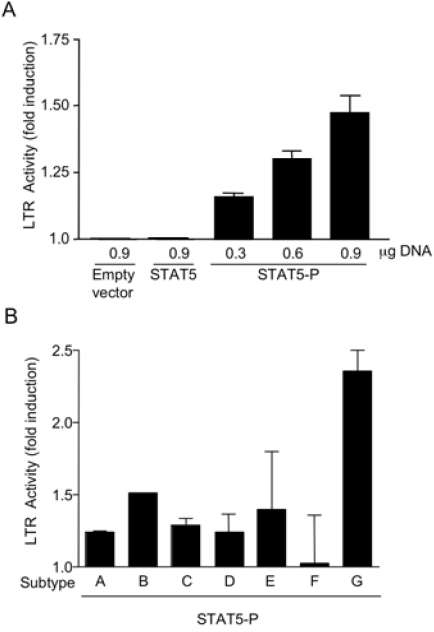
**STAT5-P transactivates the HIV-1 LTR from subtypes A through G in U937 cells with different potency. A.** U937 cells were co-transfected with HIV-1 LTR subtype B responsive firefly luc and vectors expressing either STAT5 or STAT5-P or an empty vector. The results are mean ± SEM of 3 independent experiments. **B**. U937 cells were co-transfected with vectors expressing the firefly luc under the control of HIV-1 LTR of subtypes A through G and either empty vector or STAT5-P. The luc activity of different HIV-1 LTR subtypes in the presence of vectors expressing either STAT5-P or control empty vector (to which the arbitrary value of 1 was assigned) is shown as fold increase. The results are mean ± SEM of 2 independent experiments.

Selliah *et al.* have shown that the γc-cytokines IL-2 was able to phosphorylate STAT5 and transactivate the HIV-1 LTR [15]. However, both IL-2 and other STAT5-activating cytokines are known to activate multiple pathways that may influence HIV transcription and expression [27]. Therefore, we next investigated whether STAT5 phosphorylation *per se* could directly lead to LTR transactivation by use of different STAT5 expression vectors. In particular, a reporter plasmid carrying the firefly luciferase gene under the control of the β-casein promoter was transfected in 293T cells together with the pXM vector expressing either a constitutively phosphorylated isoform of STAT5 (pXM-HA-STAT5-P) or a conventional, unphosphorylated STAT5 (pXM-HA-STAT5A); the pXM empty vector alone was also included as control. These vectors were tested for their capacity to induce transcription of a STAT5 physiological target such as the β-casein promoter [23]. As predicted, STAT5-P, but not STAT5 or transfection of the empty vector, transactivated the β-casein promoter (Fig. **2A**). Next, we investigated whether STAT5-P could modulate transcription directed by the subtype B HIV-1 LTR. 293T-LTR-GFP cells, carrying a stably integrated HIV-based lentiviral vector, expressing GFP under the control of the 5’ LTR, were transfected with an expression plasmid containing either STAT5-P or STAT5. STAT5-P, but not STAT5, increased the level of GFP^+^ cells in a concentration-dependent manner, as detected 48 h after transfection (Fig. **2B**). This effect was reproduced in promonocytic U937 cells transiently co-transfected with an HIV-LTR-luc reporter gene (Fig. **3A**). Overall, our findings confirm and extend those of Selliah *et al.* [15] in primary CD4^+^ T cells indicating that activation of STAT5 *per se* can trigger HIV transcription in different cell types.

### Constitutively phosphorylated STAT5 transactivates the LTR of HIV-1 subtypes A through G in U937 cells with different potency.

U937 cells were transfected with vectors expressing the luc reporter gene under the control of LTR sequences of HIV-1 subtypes A through G together with an expression vector carrying the STAT5-P gene or with an empty vector as control. STAT5-P transactivated the LTR of all different subtypes, except F, although with different potency. The highest transactivation capacity was observed in the presence of the subtype G LTR (Fig. **3B**), containing a perfect STAT binding site (Table **1**). Of interest is the fact that no STAT5-P mediated transactivation was observed on the subtype F LTR characterized by the lowest matching score for STAT consensus binding sites (Table **1**).

### GM-CSF promotes STAT5 binding to the subtype G HIV-1 LTR in U937 cells.

**Fig. (4):**
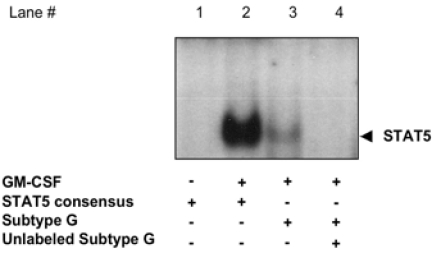
**GM-CSF activates STAT5 binding to the HIV-1 LTR subtype G in U937 cells**. WCE from either unstimulated or GM-CSF stimulated U937 cells were incubated with radiolabeled oligonucleotides. A 100-fold excess of unlabeled LTR-subtype G was added in lane 4.

In order to confirm that physiological activation of STAT5 could lead to specific DNA binding to HIV LTR sequences, we stimulated U937 cells with the βc-cytokine GM-CSF for 15 min. WCE from either unstimulated or GM-CSF stimulated U937 cells were incubated with radiolabeled oligonucleotides corresponding to either a STAT5 consensus probe, to the regions corresponding to either the HIV-LTR STAT consensus sequence of subtype G. A DNA-binding complex was readily observed by EMSA with WCE from GM-CSF stimulated U937 incubated with the subtype G STAT consensus sequence probe (Fig. **4**, lane 3). This complex was specific in that it was competed with a 100-fold excess of unlabeled subtype G STAT consensus probe (Fig. **4**, lane 4).

## DISCUSSION

In the present study, we have analyzed the LTR promoter regions of HIV-1 subtypes A through G and G”(CRF02-AG) for their potential capacity to bind STAT5, a transcription factor induced by cell stimulation with several γc- and βc-cytokines. We observed heterogeneity in these putative STAT-binding sites among different subtypes with one sequence from subtype G perfectly matching the STAT binding consensus sequence. Other subtype G as well as B, C and D putative STAT-binding sequences showed a significant similarity with a canonical STAT binding consensus site whereas subtype A, E and F did not predict a STAT-binding site. STAT5 binding to the perfectly matching subtype G sequence was associated with a higher transactivation capacity of a constitutively phosphorylated STAT5 (STAT5-P) in U937 cells. In addition to U937 cells, STAT5-P transactivated the subtype B HIV-LTR in stably transfected 293T cells.

The presence of 3 different STAT-consensus elements (named S1, S2 and S3) in the subtype B LTR of NL4-3 was previously reported by independent investigators [15]. In addition, we have recently confirmed the presence of S2 and S3 located in positions -137 to -130 and -85 to -77, respectively [18]. In particular, the STAT binding consensus sequence of the HIV-1 LTR of subtype B here described coincides with the S3 sequence reported by Selliah *et al.* [15], whereas the S2 site, located at -137 to -130, is present only in the subtype B LTR and was not detected in others subtypes by both the bioinformatics softwares used in this study. Selliah *et al.* provided the first evidence that cytokine-mediated activation of STAT5 [28] could lead to increased HIV transcription and viral expression [15]. However, cytokine stimulation can also trigger different pathways leading to increased HIV transcription, including NF-κB and MAP-kinase activation [29-31], as reviewed [27]. In particular, both IL-6 and GM-CSF stimulation of U1 cell lead to activation of estrogen-receptor kinase-1/-2 (ERK-1/-2) turning on Jun/Fos (AP-1) [32, 33], a transcription factor previously linked to the activation of HIV transcription [34-38]. Here, we provide direct evidence that activated STAT5 *per se*, independently of γc- or βc-cytokine stimulation or the activation of parallel signaling pathways, leads to LTR transactivation. In addition, we demonstrate that constitutively phosphorylated STAT5 transactivates the LTR of the different subtypes with different potency, with the highest levels observed with the subtype G LTR containing a canonical STAT binding consensus sequence as demonstrated by EMSA. The presence of 2 putative STAT-binding site in the LTR of subtype B [18] may support the observation that higher levels of transactivation were observed in comparison to cells transfected with LTR of subtypes C and D, showing matching scores similar to that of subtype B. Finally, constitutively activated STAT5 did not transactivate the LTR of subtype F that showed the lowest matching score for STAT consensus binding site.

The increasing prevalence of HIV-1 transmission through heterosexual contacts and the growing number of immigrants from non-Western countries, where non-B subtypes and recombinant forms are prevalent, suggest the possible emergence in Europe of a new epidemic wave of HIV-1 non-B subtypes as well as recombinant forms [3]. Three A-family subtype and two into the G subtype have been reported in Italy [39, 40]. The 5 non-B-subtype HIV-1 isolates have been identified among 23 variants (prevalence, 21.74%) isolated during the 2000 to 2001 period in heterosexuals [39]. More recently, Camacho and colleagues reported that the commonest subtypes in portugal is B (41.7%), but the subtype G account for 29.4%, while other non-B subtypes rated 12.8% and recombinant forms represented 16.1% of the samples [41]. In this context, the heterogeneity in STAT binding site could be relevant for an increasing fraction of HIV infected individuals worldwide.

STAT5 is a key transcription factor activated by several cytokines [16, 17] and it is a critical component of the IL-2 receptor-mediated signal for CD4^+^ T cell proliferation and activation [42]. Therefore, the observation of its differential effect on the transcription of different subtypes could be relevant in term of pathogenicity. In addition, several other γc-cytokines activating STAT5, including IL-2, IL-7, and IL-15, have been implicated as regulators of HIV replication [43, 44]. Our results expand the observation of a positive effect of STAT5 activation on subtype B viral transcription [15] to clades A through G. In this regard, we have recently reported that a constitutively activated, C-terminus truncated STAT5 isoform (STAT5Δ) is frequently detected in leukocytes of HIV^+^ individuals and provided direct evidence that this post-translationally modified STAT plays a suppressive rather than an inductive role on viral transcription and virus expression [18]. However, no information is available on whether individuals infected with HIV-1 subtypes other than B show a similar “aberrant” profile of constitutive STAT5Δ activation.

In conclusion, heterogeneity of STAT5 binding sequence is a novel element distinguishing different HIV-1 subtypes. Although there is no evidence at present for subtype specific variation in virulence or transmission our findings support the possibility that sequence differences among the subtypes, at least in the LTR region, can result in diverse biological properties and pathogenic potential [11, 12, 45].

## Figures and Tables

**Table 1:** Genomatrix Analysis of STAT Consensus Sequence

HIV-LTR Subtype	Core Sequence Similarity*	Matrix Sequence Similarity**
A	0.758	0.874
B	1	0.742
C	1	0.768
D	1	0.742
E (CRF01-AE)	0.758	0.874
F	Under threshold	Under threshold
G	1	1
G” (CRF02-AG)	1	0.761

* Defined as the consecutive (usually 4) highest conserved positions of the matrix. The maximum core similarity of 1.0 is only reached when the highest conserved bases of a matrix match exactly in the sequence.

** Defined as the entire sequence of the putative DNA binding site. A perfect match to the matrix is indicated by a score of 1.0 (each sequence position corresponds to the highest conserved nucleotide at that position in the matrix), a statistically significant match to the matrix must show a similarity of >0.8.

**Table 2:** Intra-Subtype Heterogeneity of STAT Consensus Sequences

HIV-LTR Subtype	Putative STAT binding sequence	No. of Sequences/Total	%
A	TTTCCAGGGGAG	24/39	61.5
B	TTTCCAGG_GAG	35/54	64.8
C	GTTCCAG__GAG	46/75	61.3
D	TTTCCAG_GGAG	21/40	52.5
E (CRF01-AE)	TTTCCAGGGGAG	29/30	96.6
F	TTTCCAGAGGGC	5/9	55.5
G	TTTCC__GGGAA	1/28	3.5
G” (CRF02-AG)	TTTCC_GGGGAG	14/19	73.6
